# Production of Antifungal Chitinase by *Aspergillus niger* LOCK 62 and Its Potential Role in the Biological Control

**DOI:** 10.1007/s00284-012-0208-2

**Published:** 2012-08-26

**Authors:** Maria Swiontek Brzezinska, Urszula Jankiewicz

**Affiliations:** 1 Department of Environmental Microbiology and Biotechnology, Institute of Ecology and Environmental Protection, Nicolaus Copernicus University, Gagarina 9, Toruń, Poland; 2 Department of Biochemistry, Warsaw University of Life Sciences, Nowoursynowska 159, Warsaw, Poland

## Abstract

*Aspergillus niger* LOCK 62 produces an antifungal chitinase. Different sources of chitin in the medium were used to test the production of the chitinase. Chitinase production was most effective when colloidal chitin and shrimp shell were used as substrates. The optimum incubation period for chitinase production by *Aspergillus niger* LOCK 62 was 6 days. The chitinase was purified from the culture medium by fractionation with ammonium sulfate and affinity chromatography. The molecular mass of the purified enzyme was 43 kDa. The highest activity was obtained at 40 °C for both crude and purified enzymes. The crude chitinase activity was stable during 180 min incubation at 40 °C, but purified chitinase lost about 25 % of its activity under these conditions. Optimal pH for chitinase activity was pH 6–6.5. The activity of crude and purified enzyme was stabilized by Mg^2+^ and Ca^2+^ ions, but inhibited by Hg^2+^ and Pb^2+^ ions. Chitinase isolated from *Aspergillus niger* LOCK 62 inhibited the growth of the fungal phytopathogens: *Fusarium culmorum*, *Fusarium solani* and *Rhizoctonia solani*. The growth of *Botrytis cinerea*, *Alternaria alternata*, and *Fusarium oxysporum* was not affected.

## Introduction

Chitinases (E.C. 3.2.1.14.) are enzymes that are capable of hydrolyzing chitin to its oligomers and/or monomers. Chitinase from various origins has different enzymological properties and usually constitutes a complex chitinolytic enzyme. These enzymes play an important role in the nutrition and parasitism of bacteria and fungi. They are also involved in fungal morphogenesis and autolysis [9, 26, 27]. In recent years, the search for microorganisms antagonistic toward fungi has intensified due to the fact that they are causative factors of many plant diseases. This is usually associated with the production of antifungal compounds and extracellular hydrolytic enzymes (chitinase and 1,3-β-glucanase) [21]. Chitinolytic enzymes are able to lyse the cell wall of many fungi. The microorganisms that produce these enzymes chitynolytic enzymes are able to destroy the cell wall of many fungi. The microorganisms that produce these enzymes are capable of eradicating fungal diseases that are a problem for global agricultural production. Molds are among the most aggressive plant pathogens. They are routinely combated using chemical fungicides. However, the excessive use of these compounds, which has increased almost threefold over the past 40 years, has led to problems related to contamination and degradation of the natural environment. These substances can be lethal to beneficial insects and microorganisms in the soil, and may also enter the food chain [5].

Some mold species which produce very strong endochitinases—*Trichoderma harzianum* and *Fusarium chlamydosporum—*are of particular significance, when it comes to plant protection. Chitinase produced by bacteria often presents antagonistic activity against phytopathogenic fungi. Chitinase encoding genes were reported, cloned, and characterized in bacteria [29], yeast, plants, and fungi. Among the chitinolytic fungi, the best-known are the fungicidal properties of *Trichoderma harzianum*. Chitynolytic microorganisms may be an alternative to chemical agents and could be employed as natural plant protection methods against fungal diseases. To this end, microorganisms able to synthesize compounds that naturally inhibit the growth of phytopathogenic fungi are being searched for. Compared to synthetic fungicides, they do not contaminate the environment, this being a crucial factor in increased interest in the use of biological methods to combat plant pathogens.

The aim of the present study was to determine the potential of *Aspergillus niger* LOCK 62 chitinase. The chitinase was purified and characterized, and its thermal stability was investigated. The antifungal activity of the crude and purified chitinase was also tested.

## Materials and Methods

### Microorganisms

The microorganism studied was *Aspergillus niger* LOCK 62, which was obtained from the Institute of Biotechnology and Antibiotics in Warsaw (Poland). The culture was maintained on Czapek Dox medium (Difco) slants supplemented with 10 g/l of colloidal chitin, sub-cultured regularly every 2 weeks and stored at +4 °C. Spore suspension was prepared by agitation of Czapek Dox cultures with a 0.1 v/v% solution of Tween 80 up to a concentration of 10^7^ spores/ml.

Eight pathogenic fungi were used as indicator strains: *Alternaria alternata* (isolate from kohlrabi), *Fusarium oxysporum* (isolate from potato), *Fusarium solani* (isolate from parsley), and *Botrytis cinerea* (isolate from tomato). All fungi were from the Bank of Plant Pathogens in Poznan. The studies also included *Fusarium culmorum* (isolate from pine) and *Rhizoctonia solani* (isolate from pine). These two plant pathogens were purchased from the Faculty of Forestry, Agricultural University in Poznan.

### Medium and Culture Conditions

The spore suspension was inoculated into 500 ml of liquid medium containing different carbon and nitrogen sources. Composition of medium 1 was: 0.3 % NaNO_3_; 0.1 % KH_2_PO_4_; 2 % saccharose; 0.05 % KCl; 0.05 % MgSO_4_·7H_2_O; and 0.001 % FeSO_4_·7H_2_O. The pH of the medium was adjusted to 6.5. The composition of medium 2 was: 0.05 % KH_2_PO_4_, 0.05 % K_2_HPO_4_, 0.003 % MgSO_4_·7H_2_O, and 0.15 % yeast extract. The pH of the medium was adjusted to 6.5. To each medium, a single substrate was added for microbial chitinase production (2 % shrimp shell waste, crab shell powder chitin, or colloidal chitin). Cultivation was at 26 °C for 10 days with shaking (100 rpm) and then cultures were centrifuged at 10,000×*g* for 10 min at +4 °C. Colloidal chitin was prepared using the method of Lingappa and Lockwood [17]. The shrimp shell waste was purchased by Krymar facility in Iłow. Chitin powder from shell crab was purchased from Sigma-Aldrich. All experiments were conducted in triplicate.

### Chitinase Activity

The activity of chitinase in the supernatant was determined using the synthetic fluorogenic substrate 4-methylumbelliferyl *N*-acetyl-β-d-glucosaminide (4MU-GlcNac) (Sigma-Aldrich) [10, 19]. The reaction mixture contained: 1 ml crude chitinase, 0.125 ml substrate 4MU-GlcNac solution (the final concentration in sample was 50 μM/l) and 0.125 ml of phosphate buffer (50 mM, pH 7). The control, prior to addition of the substrate, was treated with 0.1 ml solution of HgCl_2_ to deactivate the enzymes present in the sample (final concentration: 4 mM/l). The mixture was incubated in the dark for 1 h at the temperature of 40 °C. After incubation, enzymatic reactions were stopped by adding HgCl_2_. The released methylumbelliferone (MU) was measured fluorimetrically at 318 nm excitation and 445 nm emission using Hitachi F 2500 spectrofluorometer. In order to determine the optimum pH of enzyme activity different buffers were used (pH 4–8) at 50 mM concentrations. The enzyme activity (*U*) was defined as nM MU released per ml per h.

### Purification of Chitinase

All purification procedures were carried out at 4 °C. After cultivation of the *Aspergillus niger* LOCK 62 isolate in medium 2 containing colloidal chitin for 6 days, the cells were removed by centrifugation at 10,000×*g* for 20 min. Chitinase was purified by a two-step purification involving ammonium sulfate precipitation and chitin affinity chromatography.

Fractionation with ammonium sulfate: The supernatant (2 l) was precipitated using ammonium sulfate to 85 % saturation. The protein deposit was obtained by centrifugation (16,000×*g*, 30 min), dissolved in 50 mM sodium phosphate buffer (pH 7.0) and dialyzed against the same buffer overnight.

Chitin affinity chromatography was done according to the modified method of Escott et al. [7]. The same volume of 1 % (w/v) colloidal chitin in 50 mM sodium phosphate buffer (pH 7.0) was added to desalted enzyme solution and incubated for 2 h at 4 °C. This solution was centrifuged (10,000×*g*, 15 min), then the supernatant was discarded, and the deposit was washed two times with 50 mM sodium phosphate buffer (pH 7.0). The bound proteins were eluted with 50 mM acetate buffer, pH 4.0. The obtained preparations were centrifuged and dialyzed overnight. The thus obtained enzyme solution was used for further research. After each purification step, the activity of chitinases and protein content was determined.

### Determination of Protein Concentration

Protein concentration was determined using the method of Bradford [4] with bovine serum albumin as a standard.

### Polyacrylamide Gel Electrophoresis

Electrophoresis under denaturing conditions (SDS-PAGE) was performed in a 12 % polyacrylamide gel according to the procedure described by Laemmli [15] in Tris–Glycine buffer pH 8.3. The protein bands were visualized using Coomassie Brilliant Blue R-250.

### Determination of Molecular Mass of the Enzyme

The molecular mass of the enzyme was estimated by SDS-PAGE. The molecular weight standards were used: phosphorylase b (97 kDa), bovine serum albumin (66 kDa), ovalbumin (45 kDa), carbonic anhydrase (31 kDa), soybean trypsin inhibitor (21 kDa), and lysozyme (14 kDa).

### Characteristics of Crude and Purified Chitinase

The optimum temperature activity of crude and purified chitinase was determined in the temperature range from 40 to 70 °C. The thermal stability of crude and purified chitinase was determined. The enzyme was initially pre-incubated at various temperatures (40, 50, and 60 °C) and at different time intervals (0, 30, 60, 90, 120, and 180 min). After the heat treatment, samples were cooled and assayed for residual activity at 40 °C. The optimum pH was determined in the range from 4.0 to 8.0. The buffer systems were as follows: 50 mM acetate buffer for the pH 4–5 range and 50 mM sodium phosphate buffer for the pH range of 6–8. The effect of metal ions on activity was determined following pre-incubation of crude and purified chitinase for 30 min at 4 °C in the presence of divalent metal ions (Mg, Ca, Hg, Zn, Mn, Cd, Pb) in final concentration 1 mM, after which the substrate was added and the residual activity tested.

### Antifungal Activity

Crude and purified chitinase were tested for inhibitory activity against the growth of the fungal strains: *Alternaria alternata, Fusarium oxysporum, Fusarium solani, Fusarium culmorum, Botrytis cinerea*, and *Rhizoctonia solani*. The antifungal activity of chitinase was estimated using a growth inhibition assay described earlier [30]. Antifungal activity was observed directly on petri plates of Czapek Dox (Difco) medium where the test microorganisms were placed with crude and purified chitinase on (experimental group) and without chitinase (control). Both groups were incubated for 72 h at 25 °C. The inhibition ratio was calculated according to the formula [30]:

$$ {\text{Inhibition ratio }}\left( \% \right) = (C - E)/C \times 100\,\% $$

where *C* is the average diameter of colonies in the control, *E* is the average diameter of colonies in the experimental group. All experiments were conducted in triplicate.

In this study, we adopted certain inhibition criteria: no inhibition (–; 0–20 %), moderate inhibition (+; 21–30 %), strong inhibition (++; 31–50 %), and very strong inhibition (+++; >50 %).

## Results and Discussion

**Fig. 1:**
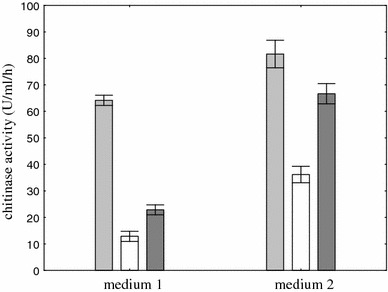
Effect of composition of medium on chitinase production. To each medium, one substrate was added: colloidal chitin (*light-shaded square*), shrimp shell waste (*dark-shaded square*), and crab shell powder chitin (*open square*). Cultivation was performed at 26 °C for 6 days. *Vertical bars* represent standard deviation (*n* = 3)

**Fig. 2:**
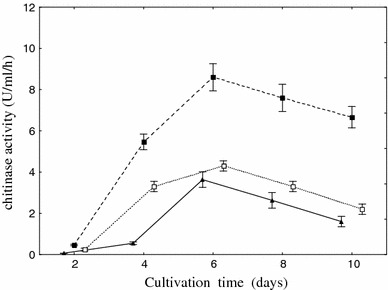
Effect of cultivation time on the chitinase production. Cultivation was performed at 26 °C on medium 2 with colloidal chitin (*filled square*), shrimp shell waste (*open square*) and crab shell powder chitin (*filled triangle*). *Vertical bars* represent standard deviation (*n* = 3)

Chitinase production from *Aspergillus niger* LOCK 62 was best in medium 2 containing yeast extract as a source of carbon and nitrogen and colloidal chitin as the substrate for enzyme production (65 nmol U ml/h on medium 1 and 82 nmol U ml/h on medium 2) (Fig. 1). Chitinase activities were checked 2 days after visible fungal growth appeared (Fig. 2), whereas maximum activity was observed after 6 days of cultivation.

The literature data indicate similar results in the case of *Aspergillus* sp. S1–13 [24]. This organism produces two endochitinases and a single exochitinase, in a solid-state culture with shrimp shellfish waste as the substrate, in a liquid culture with shrimp shellfish waste and in a liquid culture with powdered chitin after 7 days of incubation [23]. Fourteen *Penicillium* strains were tested on wheat bran–crude chitin mixture medium for extracellular chitinase production in solid-state fermentation. Under the experimental conditions, *Penicillium aculeatum* NRRL 2129 (= ATCC 10409) was selected as the best enzyme producer. The optimum incubation period for chitinase production by the potent organism was 72 h [3]. Lee et al. [16] showed that the highest chitinase activity from *Penicillium* sp. LYG 0704 was observed on the third day of cultivation.

**Fig. 3:**
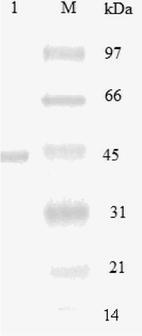
SDS-PAGE analysis of purified chitinases from *Aspergillus niger* LOCK 62. *Lane M* was loaded with standard marker mixture and *lane 1* was loaded with the purified enzyme

Summary purification steps for chitinase from *Aspergillus niger* LOCK62 are presented in Table 1. Ammonium sulfate precipitation and chitin affinity adsorption resulted in 2.3-fold purified enzyme preparation with high recovery of activity of 29 %. The specific activity of the purified enzyme was 22.5 U mg/h. The purity of the enzyme preparation was confirmed by electrophoresis after chromatographic separation step (Fig. 3). The molecular mass of chitinase was estimated to be 43 kDa.

**Table 1:** Summary of the purification procedure for *Aspergillus niger* LOCK 62 chitinase

Step	Total protein (mg)	Total activity (U)	Specific activity (U/mg)	Yield (%)	Purification factor
Crude enzyme	132	1250	9.46	100	1
NH_4_SO_4_ (85 %) dialysis	46	786	16.9	63	1.7
Affinity adsorption chromatography	16	360	22.5	29	2.3

Binod et al. [3] showed for *Penicillium aculeatum* NRRL 2129 that the yield of chitinase from SSF culture filtrate was 60.3 % and the purification factor was 2.9. The molecular weight of the purified enzymes was estimated as 82.7, 44.6, 28.2, and 26.9 kDa. Lee et al. [16] purified chitinase from *Penicillium* sp. LYG 0704 17.6-fold with an overall yield of 8.8 %. The molecular weight of the enzyme was 47 kDa. Rattanakit et al. [24] purified exochitinase from *Aspergillus* sp. S1–13 approximately 22-fold with 1 % yield. The molecular weight of the purified chitinase was 73 kDa.

**Fig. 4:**
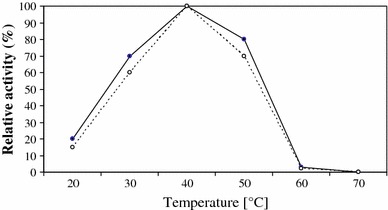
Effect of temperature on the activity of chitinase from *Aspergillus niger* LOCK 62 (crude chitinase, *filled circle*; purified chitinase, *open circle*)

In this study, the highest activity was obtained at 40 °C for both crude and purified enzymes (Fig. 4). The optimal temperature for the activity of crude and purified chitinases from *Penicillium aculeatum* NRRL 2129 and *Massilia timonae* was 50 °C [1, 3].

**Fig. 5:**
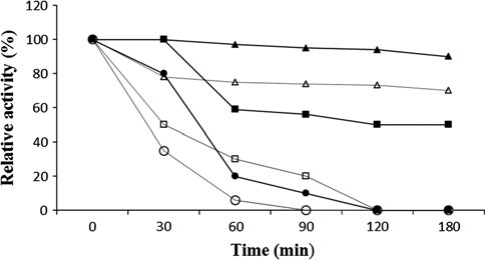
Thermal stability of crude and purified chitinase (40 °C crude chitinase, *filled triangle;* 40 °C purified chitinase, *open triangle;* 50 °C crude chitinase, *filled square;* 50 °C purified chitinase, *open square*; 60 °C crude chitinase, *filled circle*; and 60 °C purified chitinase, *open circle*)

Thermal stability was investigated at 40–60 °C (Fig. 5). After 180 min of pre-incubation of crude chitinase at 40 °C, a decrease of enzyme activity of about 10 % was observed while with purified enzyme about 25 % of activity was lost. Both the crude and the purified enzyme preparations completely lost their activity after 180 min of incubation at 60 °C. These results were similar to those observed for chitinase from other microbial sources [16, 24]. Thompson et al. [28] reported that the chitinase isolated from *Pseudomonas aeruginosa* retained 90 % of its activity up to 50 °C while another report shows that a chitinase from *Penicillium oxalium* was stable below 45 °C [25]. The crude chitinase preparation (assuming this is what the authors meant) from *Penicillium aculeatum* NRRL 2129 lost 25 % of its activity after incubation at 50 °C for 1 h [3]. Lee et al. [16] reported that the chitinase *Penicillium* sp. LYG 0704 was thermostable below the temperature of 40 °C.

**Fig. 6:**
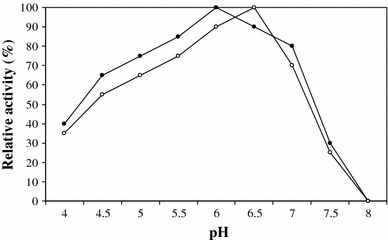
Effect of pH on the activity of chitinase from *Aspergillus niger* LOCK 62 (crude chitinase, *filled circle*; purified chitinase, and *open circle*)

The optimal pH for crude chitinase produced by *Aspergillus niger* LOCK 62 was found to be 6.5, but for purified enzyme maximum activity was at pH 6 (Fig. 6). Pritsch et al. [22] reported that four species of studied fungi showed differences in the pH optima for chitinase activity. The optimal pH for the chitinase activity of *Lactarius subdulcis* was pH 6.0, but chitinase from *Xerocomus* cf*. chrysenteron* had optimum activity at pH 5.0–5.5. *Cortinarius obtusus* and *Russula ochroleuca* showed optimum activity at pH 3.5. According to Wang et al. [31], chitinase from *Monascus purpureus* (Went) was optimally active at pH 7. On the other hand, crude chitinase from *Penicillium aculeatum* NRRL 2129 was active at pH 4.0 but maximum activity of the purified enzyme was at pH 5.5 [3].

The activity of crude and purified enzyme was stabilized by Mg^2+^ and Ca^2+^ ions. The activity of the enzyme was inhibited by Hg^2+^ and Pb^2+^ ions (Table 2). These results were similar to those observed for chitinase from other microbial sources [31]. The endochitinases from *Massilia timonae* were stabilized also by Mn^2+^, Ag^2+^, Na^2+^, and Zn^2+^ ions [1].

**Table 2:** Effect of metal ions on activity of chitinase from *Aspergillus niger* LOCK 62

Metals (1 mM/l)	Relative activity (%)	
	Crude chitinase	Purified chitinase
None	100	100
Mn^2+^	42	32
Mg^2+^	104	101
Zn^2+^	72	62
Ca^2+^	98	105
Hg^2+^	0	0
Pb^2+^	0	0
Cd^2+^	44	34

Antifungal activity of crude and purified chitinase was observed against *Fusarium culmorum, Fusarium solani*, *and Rhizoctonia solani* (Table 3). The growth inhibition of *Fusarium culmorum* was the strongest both by crude and purified enzymes (70 and 60 %, respectively). The growth of *Fusarium solani* was strongly inhibited by crude chitinase (73 %). The growth of *Botrytis cinerea, Alternaria alternata, and Fusarium oxysporum* was not inhibited.

**Table 3:** Antifungal activity of chitinase from *Aspergillus niger* LOCK 62 against various phytopathogens

Phytopathogens	Antifungal activity^a^	
	Crude chitinase	Purified chitinase
*Alternaria alternata*	**–**	**–**
*Fusarium solani*	**+++**	**+**
*Fusarium oxysporum*	**–**	**–**
*Fusarium culmorum*	**+++**	**+++**
*Botrytis cinerea*	**–**	**–**
*Rhizoctonia solani*	**+**	**+**

^a^Antifungal activity was classified as no inhibition (–; 0–20 %), moderate inhibition (+; 21–30 %), strong inhibition (++; 31–50 %), and very strong inhibition (+++; >50 %)

Joo [12] studied the antifungal activity of purified and crude chitinases produced by *Streptomyces halstedii*. Both purified and crude chitinolytic enzymes inhibited the growth of phytopathogens to some lesser or greater extent. Purified chitinases inhibited the mycelium growth of *Alternaria alternata*, *Colletotrichum gloeosporioides*, *Fusarium oxysporium*, and *Stemphylum lycopersici*, whereas unpurified chitinases were additionally inhibited by *Phytophtora capsci*. Despite the fact that many *Streptomyces* strains produce chitinases, their activities differ considerably. *Streptomyces halsteii* produces highly active chitinases and this implies that they might be used as a product for biological plant protection. Chitinolytic enzymes may reveal stronger inhibition in relation to mycelia of phytopathogens compared to commercial chitinases. Among the main fungal antagonists, the following are included: *Bacillus subtilis*, *Bacillus cereus*, *Bacillus licheniformis*, *Bacillus polymyxa*, *Bacillus amyliliquefaciens*, *and Bacillus vallismortis* [13, 14, 20, 32]. Among fungi, fungistatic properties of the genus *Trichoderma* are the most widely researched. The enzymes they produce: chitinases, proteases, and glucanases, which decompose the cell wall of phytopathogens, may find potential use in biocontrol [18]. These enzymes are strong inhibitors of many important plant pathogens. Chitinase produced by *Trichoderma* is the most widely investigated enzyme of fungicidal effects [8]. α-(1-3)-Glucanases produced by *Trichoderma harzianum* bind to the cell wall of different pathogenic fungi, including: *Aspergillus niger*, *Botrytis cinerea*, *Colletotrichum acutatum*, *and Penicillium aurantogriseum* and cause its degradation [2]. Species from the genus *Trichoderma* have been studied for many years for their usability as bio controlling factors in agriculture [11]. The chitinase from *Myrothecium verrucaria* degrades the cuticle of the mosquito *Aedes* *aegypti*. Chitinase from *Beauveria bassiana* attacks *Galleria mellonella* and *Trichoplusia ni* [6].

The studies described herein confirm the information that compounds of natural origin may significantly contribute to effectively combating plant diseases. It is therefore worthwhile to search in the natural environment for microorganisms capable of combating plant diseases and thus curtail the chemization of the environment.
